# Supplementary material for: RNA-seq reveals more consistent reference genes for gene expression studies in human non-melanoma skin cancers
Source: PeerJ. 2017 Aug 21;5:e3631. doi: 10.7717/peerj.3631 (PMC5572537; doi:10.7717/peerj.3631)

Supplmentary Figure 1 - Distribution plots of various metrices

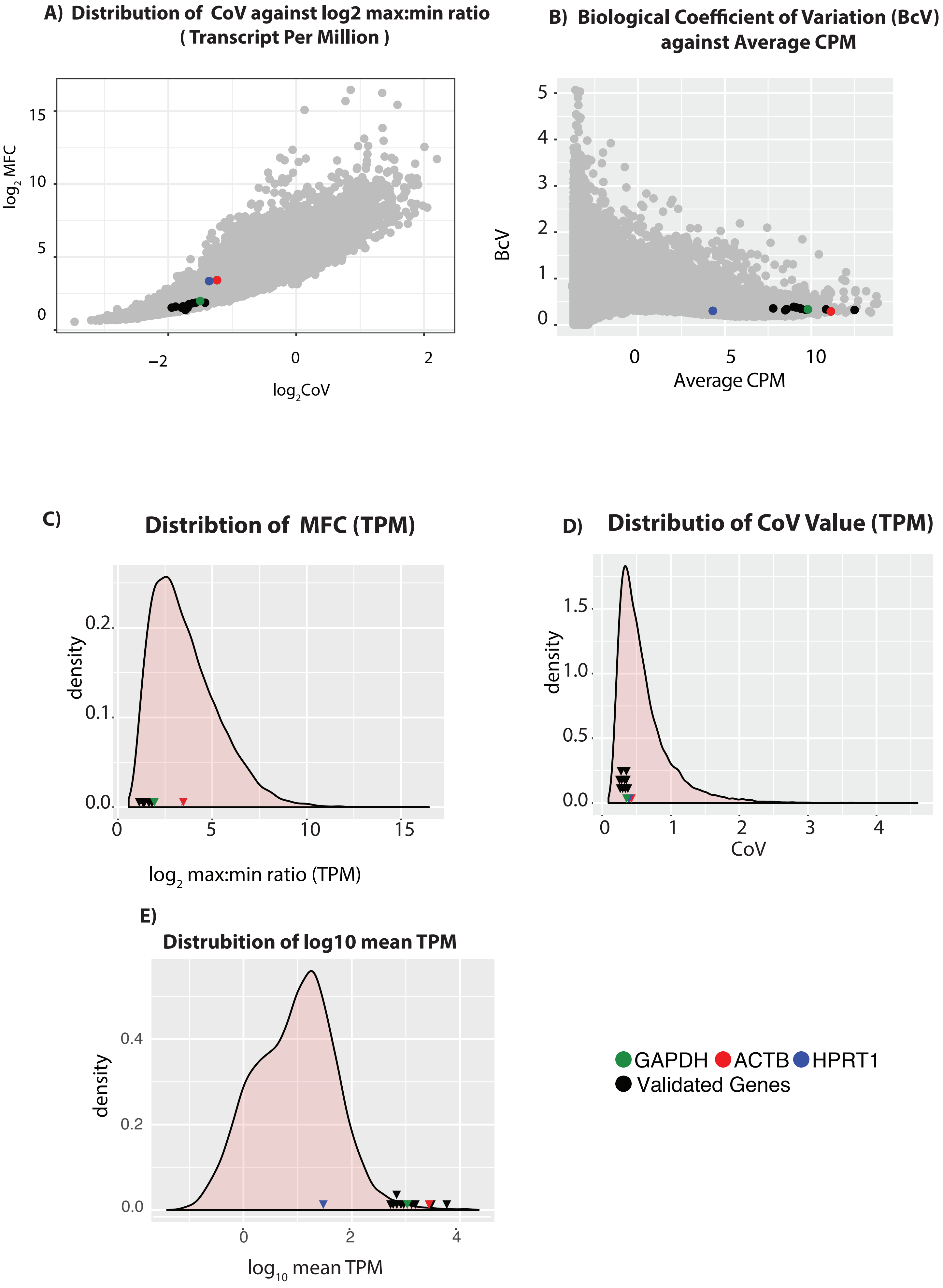

Supplement: Figure S1 — (A) Scatterplot comparing Coefficient of variation (CoV) values against max:min ratio (MFC) of gene expression quantified as Transcript per kilobases million (TPM) for each gene. (B) Scatterplot comparing BCV values with average log2CPM for each gene. (C–D) Distribution of MFC, CoV and mean TPM for each genes detected from RN-seq of patient derived skin samples. Arrows indicates the values of the candidates (black arrow) and standard housekeeping genes on the x-axis. [file peerj-05-3631-s001.pdf]
