# Supplementary material for: RNA-seq reveals more consistent reference genes for gene expression studies in human non-melanoma skin cancers
Source: PeerJ. 2017 Aug 21;5:e3631. doi: 10.7717/peerj.3631 (PMC5572537; doi:10.7717/peerj.3631)
Supplement: Figure S2 — (A) Radio buttons for users to toggle value type to be plotted on the × and y-axis. Users can choose between plotting log10 Maximum Fold Change (log10MFC), log10 Transcript Per Million (log10TPM) and Coefficient of Variation (CoV) B) Scatterplot showing data points derived from each measured transcript. Only genes with TPM > 0 for all samples are shown. Red dots represents genes selected either by user selection of points on the chart or are present in the user input (C). Green dots represent the gene with information displayed in (E) and is selected by clicking on the row in (D). (C) Input box for users to search for genes in the datasets. (D) Table showing metrics of selected genes (coloured dots) in scatterplot (E) Gene information of specific gene in the scatterplot (Green dots). [file peerj-05-3631-s002.pdf]

Supplmentary Figure 1

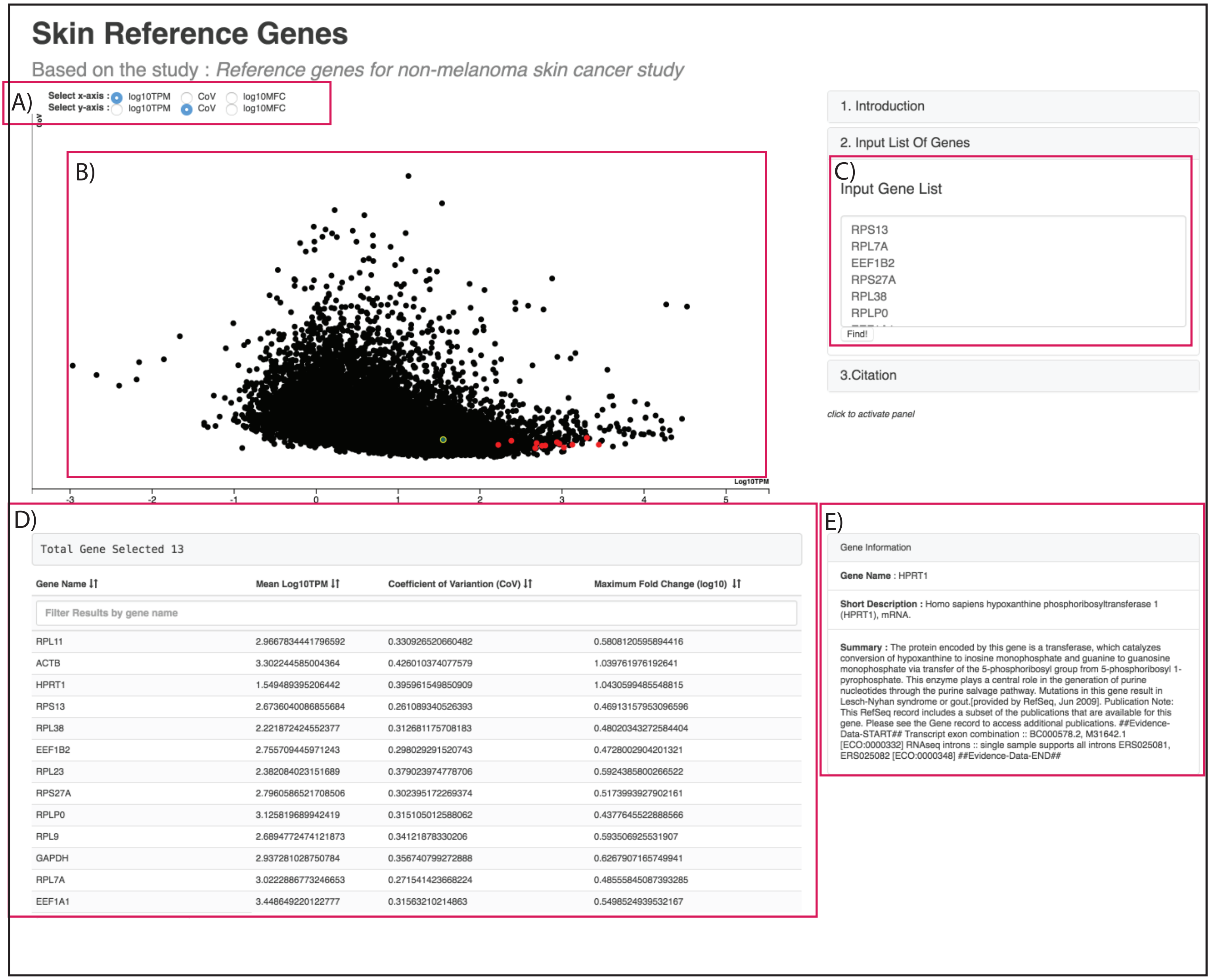

Gene Name	Mean Log10TPM	Coefficient of Variantion (CoV)	Maximum Fold Change (log10)
RPL11	2.9667834441796592	0.330926520660482	0.5808120595894416
ACTB	3.302244585004364	0.426010374077579	1.039761976192641
HPRT1	1.549489395206442	0.395961549850909	1.0430599485548815
RPS13	2.6736040086855684	0.261089340526393	0.46913157953096596
RPL38	2.221872424552377	0.312681175708183	0.48020343272584404
EEF1B2	2.755709445971243	0.298029291520743	0.4728002904201321
RPL23	2.382084023151689	0.379023974778706	0.5924385800266522
RPS27A	2.7960586521708506	0.302395172269374	0.5173993927902161
RPLP0	3.125819689942419	0.315105012588062	0.4377645522888566
RPL9	2.6894772474121873	0.34121878330206	0.593506925531907
GAPDH	2.937281028750784	0.356740799272888	0.6267907165749941
RPL7A	3.0222886773246653	0.271541423668224	0.48555845087393285
EEF1A1	3.448649220122777	0.31563210214863	0.5498524939532167

Supplementary Figure 1 - Screenshot of the online resource of reference genes for non-melanoma skin cancers. **A)** Radio buttons for users to toggle value type to be plotted on the x and y-axis. Users can choose between plotting log10 Maximum Fold Change (log10MFC), log10 Transcript Per Million (log10TPM) and Coefficient of Variation (CoV) **B)** Scatterplot showing data points derived from each measured transcript. Only genes with TPM > 0 for all samples are shown. Red dots represents genes selected either by user selection of points on the chart or are present in the user input (C). Green dots represent the gene with information displayed in (E) and is selected by clicking on the row in (D). **C)** Input box for users to search for genes in the datasets. **D)** Table showing metrics of selected genes (coloured dots) in scatterplot **E)** Gene information of specific gene in the scatterplot (Green dots).
